# Comprehensive Health Insurance and access to maternal healthcare services among Peruvian women: a cross-sectional study using the 2021 national demographic survey

**DOI:** 10.1186/s12884-023-06086-3

**Published:** 2023-11-15

**Authors:** Eduardo Ramos Rosas, Volker Winkler, Luis Huicho, Magaly M. Blas, Stephan Brenner, Manuela De Allegri

**Affiliations:** 1 Heidelberg Institute of Global Health, University Hospital and Medical Faculty, Heidelberg University, Im Neuenheimer Feld 130.3, 69120 Heidelberg, Germany; 2 Centro de Investigación en Salud Materna E Infantil, Centro de Investigación Para El Desarrollo Integral y Sostenible and School of Medicine, Universidad Peruana Cayetano Heredia, Lima, Peru; 3 School of Public Health and Administration, Universidad Peruana Cayetano Heredia, Lima, Peru

**Keywords:** Health insurance, Peru, Maternal healthcare, SIS (Seguro Integral de Salud), Women’s health, Inequities, Comprehensive Health Insurance

## Abstract

**Background:**

The government-subsidized health insurance scheme *Seguro Integral de Salud* (“SIS”) was introduced in Peru initially to provide coverage to uninsured and poor pregnant women and children under five years old and was later extended to cover all uninsured members of the population following the Peruvian *Plan Esencial de Aseguramiento Universal* – “PEAS” (Essential UHC Package). Our study aimed to analyze the extent to which the introduction of SIS has increased equity in access and quality by comparing the utilization of maternal healthcare services among women with different insurance coverages.

**Methods:**

Relying on the 2021 round of the nationally-representative survey “ENDES” (*Encuesta Nacional Demográfica y de Salud Familiar*), we analyzed data for 19,181 women aged 15–49 with a history of pregnancy in the five years preceding the survey date. We used a series of logistic regressions to explore the association between health insurance coverage (defined as No Insurance, SIS, or Standard Insurance) and a series of outcome variables measuring access to and quality of all services along the available maternal healthcare continuum.

**Results:**

Only 46.5% of women across all insurance schemes reported having accessed effective ANC prevention. Findings from the adjusted logistic regression confirmed that insured women were more likely to have accessed ANC services compared with uninsured women. Our findings indicate that women in the “SIS” group were more likely to have accessed six ANC visits (aOR = 1.40; 95% CI 1.14–1.73) as well as effective ANC prevention (aOR = 1.32; 95% CI 1.17–1.48), ANC education (aOR = 1.59; 95% CI 1.41–1.80) and ANC screening (aOR = 1.46; 95% CI 1.27–1.69) during pregnancy, compared with women in the “Standard Insurance” group [aOR = 1.35 (95% CI 1.13–1.62), 1.22 (95% CI 1.04–1.42), 1.34 (95% CI 1.18–1.51) and 1.31(95% CI 1.15–1.49)] respectively. In addition, women in the “Standard Insurance” group were more likely to have received skilled attendance at birth (aOR = 2.17, 95% CI 1.33–3.55) compared with the women in the “SIS” insurance group (aOR = 2.12; 95% CI 1.41–3.17).

**Conclusions:**

Our findings indicate the persistence of inequities in access to maternal healthcare services that manifest themselves not only in the reduced utilization among the uninsured, but also in the lower quality of service coverage that uninsured women received compared with women insured under “Standard Insurance” or “SIS”. Further policy reforms are needed both to expand insurance coverage and to ensure that all women receive the same access to care irrespective of their specific insurance coverage.

**Supplementary Information:**

The online version contains supplementary material available at 10.1186/s12884-023-06086-3.

## Background

As core elements of Sustainable Development Goal 3 (SDG3), *Ensure healthy lives and promote well-being for all,* target 3.1 aims at reducing the maternal mortality ratio to less than 70 per 100,000 live births, while target 3.2 aims at reducing the neonatal mortality to at least as low as 12 per 1,000 live births and the under-five mortality to at least as low as 25 per 1,000 live births by 2030 [1]. However, worldwide maternal- and neonatal mortality remain high despite global efforts to accomplish these goals, with about 295,000 maternal deaths and around five million neonatal deaths, according to the latest reports [2–4]. Moreover, more than 90% of these maternal- and neonatal deaths occur in low and low-middle-income countries [5, 6].

According to the World Health Organization (WHO), maternal and neonatal deaths could be substantially reduced if women had access to equitable reproductive-, maternal-, and newborn healthcare services [3, 7, 8]. Evidence has amply demonstrated that access and utilization of maternal care services of adequate quality can improve birth outcomes and hence help reduce maternal and neonatal mortality [9–11]. Furthermore, since quality maternal care services are fundamental to ensuring positive pregnancy outcomes, governments across the globe are called to ensure that they are accessible to all pregnant women, irrespective of their ability to pay [12, 13]. In turn, this emphasis on the accessibility of maternal healthcare services feeds directly into the broader discourse on Universal Health Coverage (UHC), promoted as a core overarching objective of SDG3 [14].

To extend population coverage and foster progress towards UHC, the Peruvian Government introduced in the early 2000s a government-subsidized health insurance scheme, *Seguro Integral de Salud* (“SIS” translated as “Comprehensive Health Insurance”), initially targeting pregnant women and children under five years old. The scheme was later extended to cover the poorest and all uninsured segments of the Peruvian population going beyond exclusively maternal healthcare services [15–17]. Formal workers, as well as citizens serving in the marine, police, or army, remained enrolled in the already-existing standard contributory health insurance scheme (“EsSalud”) or in the specific insurance scheme for the armed forces or police (FFAA/Police) [18]. The “SIS” possesses an exclusive physician and hospital network with around 430 hospitals and health posts distributed throughout the country, while “EsSalud” (standard contributory health insurance) provides approximately 380 healthcare centers (90 of them hospitals) distributed across the country with at least half of these hospitals located in the capital [19].

**Fig. 1:**
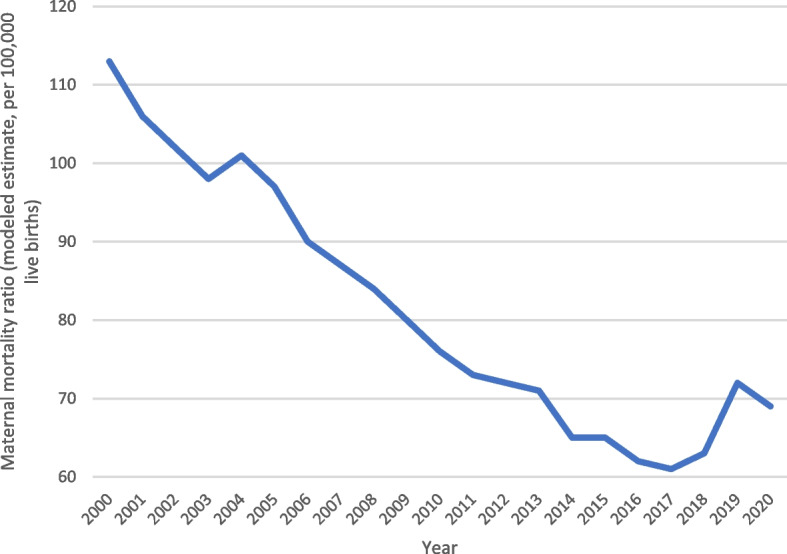
“Maternal mortality rate in Peru over the last 20 years”.

**Fig. 2:**
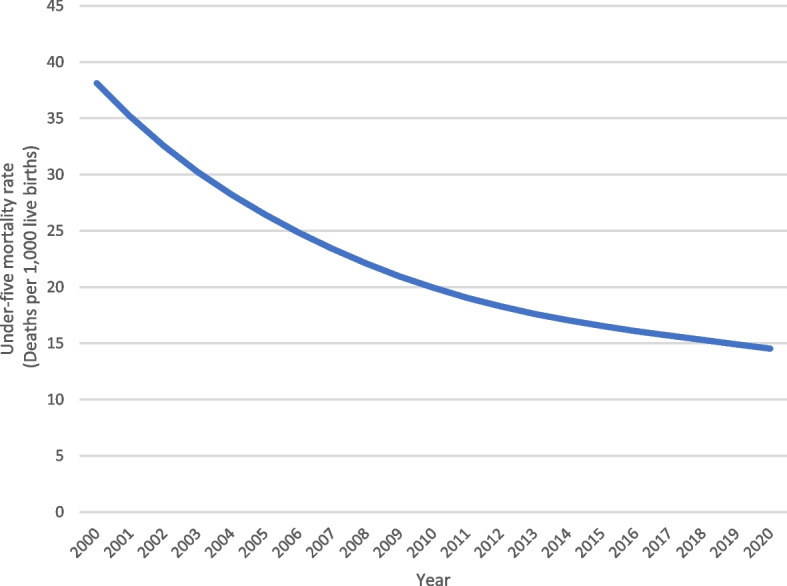
“Neonatal mortality rate in Peru over the last 20 years”.

As showed in Fig. 1 [20] and Fig. 2 [21], the maternal- and neonatal mortality rate have been decreasing in Peru over the last 20 years after the introduction of the “SIS”, fulfilling one of its mains objectives related to maternal healthcare services [22]. Nevertheless, little is known about whether women affiliated to the “SIS” have nowadays different access rates or quality of maternal healthcare services compared to women enrolled to other insurance schemes. Considering that low-income pregnant women constituted the original target group of the SIS scheme and continue to represent a vulnerable segment of the Peruvian population, understanding whether SIS-enrolled women currently have similar access rates to and quality of maternal care services is of the utmost importance because it reflects the ability of the scheme to achieve its ultimate policy objective of reaching more equitable healthcare services [23].

Our work aims to fill this existing knowledge gap by examining the access and quality of maternal healthcare services across women with different insurance coverage in Peru, specifically for year 2021. Using a wide range of maternal healthcare service indicators and drawing them from the Peruvian *Plan Esencial de Aseguramiento Universal—“*PEAS” (Essential UHC Package) guidelines [24–26], our ambition is to assess the extent to which, thanks to the “SIS”, poor, vulnerable women enrolled to this insurance really enjoy access and quality to the same care as other Peruvian women do.

## Methods

### Study design and data sources

Our cross-sectional study used data from the 2021 round of the “ENDES” (*Encuesta Nacional Demográfica y de Salud Familiar*), a nationally representative self-reporting survey designed for Peruvian women aged 15–49 years and conducted yearly by the Peruvian *National Institute of Statistics and Computing* (“INEI”) [27].

The selection of the data modules used for our study was based on the variables we were interested in, namely those that captured access to and quality of maternal healthcare services and variables that captured individual socio-demographic and economic characteristics. We merged the different data modules using the respondent’s unique identifier variable. Given our focus on maternal healthcare, out of a total of 35,847 women included in the survey, we selected exclusively those women with a history of pregnancy in the five years prior to the survey date, yielding a sample of 19,181 women [28]. Women with more than one reported pregnancy in the last five years prior to the survey’s conduction were only counted once, using their unique identification’s variable to avoid duplicates. The authors were not directly involved in data collection and obtained fully-anonymized data ready for analysis directly from the “INEI” [29].

### Variables and their measurements

Our work focuses on five outcome variables measuring access to different maternal healthcare services during the reported pregnancy period. The selection of these variables was based on point II.3.: “Pregnancy, delivery and puerperium” of the “PEAS" (Essential UHC Package) guideline, listing the recommended maternal services, which should be equally accessible to all insured women based on the *Ley de Aseguramiento Universal en Salud: Ley 29344* (Universal Health Insurance Act of 2009 in Peru) [24–26]. In addition, variables were selected based on international guideline-recommended maternal services from the WHO and Peruvian guidelines, whose fulfillment and access should provide a positive (equal) pregnancy experience for women [13, 26].

Outcome variables were dichotomized as follows: 1. Six antenatal care (ANC) visits if a woman attended at least six antenatal care visits during pregnancy following Peruvian recommendations [26], 2. Skilled attendance at birth if a woman delivered in the presence of a skilled birth attendant, considering exclusively physicians, trained nurses, or trained midwifes as such, while nursing technicians, health promoters, traditional birth attendants/midwives, relatives and friends as unskilled attendants [30], 3. Effective antenatal care (ANC) prevention if a woman attended at least six antenatal care visits during pregnancy, received iron during pregnancy, and was adequately vaccinated against Tetanus according to WHO (at least five vaccinations during her life or vaccination boost during pregnancy or two doses if vaccination status was unknown or void Tetanus) [26, 31], 4. Effective antenatal care (ANC) education if a woman attended at least six antenatal care visits during pregnancy and received advice about possible pregnancy complications during any of these visits [26, 32], and 5. Effective antenatal care (ANC) screening if women attended at least six antenatal care visits during pregnancy and a minimum of six essential clinical measures were carried out at least once during the course of the ANC visits as recommended by the “PEAS*”*: weight taken; stomach circumference measured; blood pressure measured; urine sample taken; blood sample taken; and fetus’ heartbeat heard [26]. Missing values (< 1% of the observations) were replaced as “No” in our analyses.

We categorized the explanatory variable (“Health Insurance Status”) in three groups: No insurance, SIS, and Standard Insurance. The “Standard Insurance” category includes traditional social health insurance (“EsSalud”), Armed forces/Police Health Insurance (FFFAA/Police), “EPS” (complementary private health insurance), and private insurance (PHI) [18, 33, 34]. Two factors motivate this classification: first, our wish to determine the differences in access to quality maternal healthcare services between this pro-poor insurance, the other existing ones, and the non—insurance group; and second, our wish to determine if being associated with “SIS” represents an advantage to not owning insurance at all.

To account for potential confounding, we adjusted our analysis for a number of conceptually relevant covariates, including “Age”, “Highest educational level attained”, “Ethnicity”, “Marital status”, “Type of place of residence”, “Ever had a terminated pregnancy”, “Births in last five years”, “Total children ever born”, “Total living children”, “Wealth index”, and “Household size”. In a prior analysis, we reported that these variables were significant determinants for insurance enrollment among Peruvian women and that they might influence both the type of insurance women possess and, consequently, also maternal healthcare outcomes [19]. The categorization of all outcome-, explanatory- and co-variates these variables can be found in Appendix A: Variables’ categorization.

### Analytical approach

We explored the associations between the outcome variables and insurance status through a bivariate analysis using chi-square tests. We included in Appendix B a disaggregated analysis of the outcome variables “Effective antenatal care prevention”, “Effective antenatal care education” and “Effective antenatal care screening” to assess the relationship between these sub-categories and the insurance status of Peruvian women.

The bivariate analysis between “Health Insurance Status” and our outcome variable as well as the bivariate analysis between “Health Insurance Status” and the covariates can be found as Table 1.

**Table 1:** Sample characteristics and bivariate analysis

Variable	**Health insurance status**			
***Outcome variables***				
**Six ANC visits**	**No Insurance** **N (% column)**	**SIS** **(% column)**	**Standard Insurance** **(% column)**	**Chi-square *p*-value**
*No*	436 (17.2)	1991 (14.9)	356 (10.7)	< 0.001
*Yes*	2102 (82.8)	11,327 (85.1)	2969 (89.3)	
**Skilled attendance at birth**				
*No*	110 (4.3)	741 (5.6)	22 (0.7)	< 0.001
*Yes*	2428 (95.7)	12,577 (94.4)	3303 (99.3)	
**Effective ANC prevention**				
*No*	1501 (59.1)	6977 (52.4)	1792 (53.5)	< 0.001
*Yes*	1037 (40.9)	6341 (47.6)	1533 (46.5)	
**Effective ANC education**				
*No*	773 (30.5)	3109 (23.3)	718 (21.6)	< 0.001
*Yes*	1765 (69.5)	10,209 (76.7)	2607 (78.4)	
**Effective ANC screening**				
*No*	728 (28.7)	3185 (23.9)	667 (20.1)	< 0.001
*Yes*	1810 (71.3)	10,133 (76.1)	2658 (79.9)	
***Covariates***				
**Highest educational level attained**				
Primary or no education	295 (11.6)	3103 (23.3)	123 (3.7)	< 0.001
Secondary	1125 (44.3)	7224 (54.2)	788 (23.7)	
Higher but no university	570 (22.5)	1920 (14.4)	1059 (31.8)	
Higher, university or Postgraduate	548 (21.6)	1071 (8.1)	1355 (40.8)	
**Ethnicity**				
Other	371 (14.6)	2357 (17.7)	329 (9.9)	< 0.001
Spanish	2167 (85.4)	10,961 (82.3)	2996 (90.1)	
**Marital status**				
Not currently married	548 (21.6)	240 (18.1)	481 (14.5)	< 0.001
Currently married	1990 (78.4)	10,908 (81.9)	2844 (85.5)	
**Place of residence**				
Rural	439 (17.3)	5177 (38.9)	320 (9.6)	< 0.001
Urban	2099 (82.7)	8141 (61.1)	3005 (90.4)	
**Ever had a terminated pregnancy**				
No	1976 (77.9)	10,940 (82.1)	2466 (74.2)	< 0.001
Yes	562 (22.1)	2378 (17.9)	589 (22.8)	
**Births in last five years**				
1	2225 (87.7)	11,243 (84.4)	2914 (87.6)	< 0.001
2 +	313 (12.3)	2075 (15.6)	411 (12.4)	
**Total children ever born**				
1 child	927 (36.5)	3904 (29.3)	974 (29.3)	< 0.001
2 children	803 (31.7)	4080 (30.6)	1285 (38.6)	
3 children	465 (18.3)	2550 (19.2)	705 (21.2)	
4 or more children	343 (13.5)	2784 (20.9)	361 (10.9)	
**Total living children**				
0–2	1763 (69.5)	8139 (61.1)	2290 (68.9)	< 0.001
3 +	775 (30.5)	5179 (38.9)	1035 (31.1)	
**Wealth index**				
Poorest	381 (15.0)	5062 (38.0)	147 (4.4)	< 0.001
Poorer	610 (24.0)	3946 (29.6)	520 (15.7)	
Middle	676 (26.7)	2391 (18.0)	722 (21.7)	
Wealthier	566 (22.3)	1350 (10.1)	922 (27.7)	
Wealthiest	305 (12.0)	569 (4.3)	1014 (30.5)	
**Household size**				
1–3	630 (24.8)	2910 (21.9)	614 (18.5)	< 0.001
4–6	1524 (60.1)	8196 (61.5)	2290 (68.9)	
7 +	384 (15.1)	2212 (16.6)	421 (12.6)	
**Age**	**No insurance** **Mean (SD)**	**SIS** **Mean (SD)**	**Standard Insurance** **Mean (SD)**	**Anova (*p*-value)**
15–49 (continuous)	30.84 (6.75)	29.93 (7.08)	33.65 (5.94)	< 0.001

In the final step, we performed a series of logistic regressions, one for each outcome variable, including the covariates of the bivariate analyses to adjust our results. We also adjusted the logistic regressions for regional clustering effects, including a regional variable. The compacted models can be found as Table 2. The complete models can be found in Appendix C- E.

**Table 2:** Logistic regression for “Health Insurance Status” and maternal healthcare outcomes

	**Six ANC visits**		**Skilled attendance at birth**		**Effective ANC prevention**		**Effective ANC education**		**Effective ANC screening**	
**Health Insurance Status**	*cOR*	*aOR*	*cOR*	*aOR*	*cOR*	*aOR*	*cOR*	*aOR*	*cOR*	*aOR*
*No insurance*	Ref	Ref	Ref	Ref	Ref	Ref	Ref	Ref	Ref	Ref
*SIS*	1.18 (1.05–1.32) ***	1.40 (1.14–1.73) ***	0.77 (0.63–0.94) **	2.12 (1.41–3.17) ***	1.32 (1.17–1.48) ***	1.32 (1.16–1.50) ***	1.44 (1.31–1.58) ***	1.59 (1.41–1.80) ***	1.28 (1.17–1.40) ***	1.46 (1.27–1.69) ***
*Standard Insurance*	1.73 (1.49–2.01) ***	1.35 (1.13–1.62) ***	6.80 (4.29–10.78) ***	2.17 (1.33–3.55) ***	1.24 (1.06–1.45) ***	1.22 (1.04–1.42) **	1.59 (1.42–1.78) ***	1.34 (1.18–1.51) ***	1.60 (1.41–1.82) ***	1.31 (1.15–1.49) ***

*cOR* Crude Odds-Ratio, *aOR* Adjusted Odds-Ratio for all covariates included in Table 1 (*Age, highest educational level attained, ethnicity, marital status, type of place of residence, ever had a terminated pregnancy, births in last five years, total children ever born, total living children, wealth index and household size*)

• 95% Confidence intervals in parenthesis

^•^ Significance level → *: *p* < 0.10; **: *p* < 0.05; ***p < 0.01

All statistical analysis was performed using Stata 15.1.

## Results

Table 1 summarizes the sample characteristics. Out of a total of 19,181 women included in our analysis, the majority were insured via “SIS” (*N* = 13,318, 69.5%), followed by 3,325 via “Standard Insurance” (17.3%). In comparison, 2,538 women (13.2%) were uninsured when the survey was conducted.

Approximately 86% of the women (*N* = 16,398) attended at least six antenatal care visits during pregnancy, and 18,308 women (more than 90%) reported having received skilled attendance at birth. However, while most women reported having received an effective ANC education (*N* = 14,581, 76.0%) as well as gotten an effective ANC screening during pregnancy (*N* = 14,601, 76.1%), only 8911 women (46.5%) reported to have accessed an effective ANC prevention.

The average age of respondents was 31, with a standard deviation (SD) of 7 years. Most women reported they had at least completed secondary education (80%), indicated “Spanish” as their ethnicity (84.1%), were currently married (82.1%), were urban residents (69.0%), never had a terminated pregnancy (80.2%) and had 0–2 living children (63.6%). Most women belonged to the poorest wealth index category (29.1%) and had a household size of more than three members (78.3%).

The bivariate analysis using the chi-square test of independence confirmed significant associations between our outcomes of interest and insurance status. We consistently observed that insured women (affiliated to “SIS” or to “Standard Insurance”) reported higher access rates to ANC services compared with the women in the “No insurance” group except for the variable “Skilled attendance at birth,” where the lowest access rate was reported among the “SIS” group (*N* = 12,577, 94.4%) followed by the “No insurance” group (*N* = 2428, 95.7%) and the “Standard Insurance” (*N* = 3303, 99.3%). The outcome variable “Effective ANC prevention” had the lowest access rate with only 6,341 women (47.6%) within the “SIS” group, followed by 1,533 (46.5%) in the “Standard Insurance” group and 1,037 (40.9%) in the “No insurance” group.

Findings from the adjusted logistic regression (Table 2) confirmed that insured women (both “SIS and “Standard Insurance”) were more likely to have accessed ANC services compared with uninsured women. Our findings indicate that women in the “SIS” group were more likely to have accessed six ANC visits (aOR = 1.40) as well as an effective ANC prevention (aOR = 1.32), ANC education (aOR = 1.59) and ANC screening (aOR = 1.46) during pregnancy compared with women in the “Standard Insurance” group (aORr = 1.35; 1.22; 1.34 and 1.31 respectively). However, our findings indicate that women in the “Standard Insurance” group were more likely to have received skilled attendance at birth (aOR = 2.17) compared with the women in the “SIS” insurance group (aOR = 2.12), holding all other variables constant.

## Discussion

Our study makes an important contribution to the existing literature by being one of the few studies looking specifically at the interface between health insurance status, access to and quality of maternal healthcare services in Peru. Using the most recent data we provide an accurate picture of potential maternal healthcare inequalities despite all government efforts toward reaching UHC. More broadly, our study contributes to the limited literature having examined the role of “SIS” in advancing the UHC objectives of favoring equitable access to care and expanding social health protection among the Peruvian population [19, 35].

The first remarkable finding of our study is that having health insurance increases the likelihood of women accessing maternal healthcare services compared with uninsured women. The odds ratios in the “SIS” and “Standard Insurance” groups were higher compared with the odds ratios of the uninsured group for all our ANC measurements (“Six ANC visits”, “Skilled attendance at birth”, “Effective ANC prevention”, “Effective ANC education” and “Effective ANC screening”). Without being able to establish any causal relationship between insurance status and access to healthcare services given the nature of the data at our disposal, our findings provide additional evidence that the Peruvian situation regarding maternal healthcare services is aligned with that of other Latin American countries, where the introduction of government-financed pro-poor health insurance schemes has helped to achieve high levels of maternal care coverage even among the poorest or lower educated members of the population, making reproductive health services more equitable [17] and reducing maternal and child mortality over the last two decades [35]. Nonetheless, it is still worrisome that more than 50% of the women included in our survey did not have access to effective ANC prevention. Furthermore, around 25% neither received an effective ANC education nor an effective ANC screening during pregnancy despite the availability of multiple insurance schemes in the country that should fully provide and cover maternal healthcare services [18, 24]. Specifically, the lowest rates were reported among the “No Insurance” group, reflecting that the lack of insurance coverage is associated to limited access to maternal care services as the literature reports [36, 37]. This, in turn, puts these women at more risk of developing pregnancy complications and of facing death [9, 11]. Increasing insurance coverage among women could improve maternal and newborn health outcomes. In this context, it might be helpful that the Peruvian Government analyses the availability of trained staff, bed capacity, and availability of surfactants and respirators across the country. The literature reports, that, for example, in Brazil, these factors have helped to reduce significantly neonatal mortality rate in addition to only increasing population coverage [38].

Besides, factors such as a lack of awareness of maternal healthcare benefits, transportation-related barriers to health facilities, and a lack of women’s and families’ awareness of the benefits of antenatal care visits despite their knowledge of antenatal care availability have been reported to be associated with reduced access to ANC services even when available [39]. Therefore, we encourage further analysis to find the determinants of limited access to these maternal healthcare services in the country.

We note that, despite the high access rates to maternal healthcare services among Peruvian-insured women, those in the “Standard Insurance” group were more likely to have received skilled attendance during delivery compared with women enrolled to “SIS”. Although the “PEAS” has the objective to provide equal maternal services to all insured Peruvian women [25], our results are in line with the literature suggesting that receiving skilled attendance during delivery is still skewed toward better educated women, urban residents and women belonging to the upper wealth index categories [17]. In a previous study, we found that women in the “Standard Insurance” group were more likely to present these characteristics, while women in the “SIS” group were more likely to be less educated, poorer, indigenous and more likely to live in rural areas [19]. These socioeconomical and cultural differences represent a barrier to provide equal healthcare services and also influence the preferences of Peruvian women regarding maternal and childbirth practices [40].

Our study shows that the “SIS” provides high access rates to the selected maternal healthcare services and a better quality compared to being uninsured. However, to reach UHC and overcome inequities in maternal healthcare, the Peruvian government needs to solve three main challenges: first, provide coverage to those uninsured women by targeting and enrolling them in one of the available insurance schemes [17, 19]. Second, identify the factors associated with limited access to maternal care services despite their availability in the country (specially) among insured women [24–26]. And third, ensure that better quality maternal healthcare services are available for all insured and uninsured women and not exclusively for the wealthiest or best educated either by increasing the number of trained persons in all insurance groups (but specially in the “SIS” group) or by increasing the amount of pooled finances in the current context of multiple co-existing insurance pools [41]. Considering that skilled attendance at birth is probably the most important factor in preventing maternal deaths, it is essential that it is equally available to all women independent of their socioeconomical status, region of origin, and ethnicity to further reduce the number of maternal deaths [42–44]. For example, in the Peruvian rural Amazon of the Loreto region, 70% of women give birth at home and only 7% of these deliveries are done with a skilled birth attendant [45].

### Methodological considerations

Despite of the strength of having a large sample size at a national level, our study has some weaknesses. First, as we relied on secondary data, our analysis was constrained by the variables available in the original survey. Information related to postnatal care or other maternal healthcare interventions for common physiological symptoms (such as nausea, vomiting, leg cramps or obstipation) was not available in the dataset, reducing the analysis to only five indicators, which do not necessarily cover all the relevant information related to maternal healthcare. Besides, our study does not provide information related to other relevant information such as healthcare facility distance, an important factor that might limit or reduce access to healthcare services. Consequently, we are unable to know if non-insurance enrollment is related to a lack of physical access to (maternal) healthcare services. Second, we did not include data about the number of women enrolled in the “JUNTOS” program (which provides conditional monetary support to women living in extreme poverty if they use maternal and child health preventive and curative services) [46], which might have significantly influenced the high access rates to maternal healthcare services. Third, by performing a cross-sectional study, we were only able to establish an association between health insurance status and access to maternal healthcare services, but not a causal inference to these access rates. In addition, we were not able to know if the insurance status of Peruvian women has changed across the years, so it might be possible that some women delivering before year 2021 were enrolled back then to a different insurance scheme they had in 2021. We strongly recommend further (longitudinal) studies to determine the causality of maternal healthcare access rates in order to better understand the reasons behind these inequities.

## Conclusions

Our study shows that women affiliated to the government-subsidized health insurance scheme “SIS” and to a “Standard Insurance” are more likely to access maternal healthcare services compared with uninsured women. Despite the similarity in access to maternal healthcare services among all available insurances, our findings indicate the persistence of inequities in access to maternal healthcare services manifested not only in reduced utilization among the uninsured, but also in the lower quality of service coverage among them compared to being insured. Further policy reforms are needed both to expand insurance coverage and to ensure that all women receive the same access to care irrespective of their specific insurance coverage and further research is also encouraged to understand the reason behind the limited access to maternal healthcare services, despite of their availability .

### Supplementary Information

**Additional file 1. Appendix A.** Variables’ categorization.** Appendix B.** Disaggregated bivariate analysis for “Effective ANC- prevention, -education and -screening”.** Appendix C.** Logistic regression for “Six ANC visits” and ANC 6 and “Skilled attendance at birth”.** Appendix D.** Logistic regression for “Effective ANC prevention” and “Effective ANC education”.** Appendix E.** Logistic regression for “Effective ANC screening”.

## Data Availability

The datasets analyzed during the current study are available in the INEI repository and are freely accessible under https://proyectos.inei.gob.pe/microdatos/.

## Abbreviations

SDG: Sustainable Development Goals
WHO: World Health Organization
UHC: Universal Health Coverage
SIS: “Seguro Integral de Salud”
FFAA/Police: Marine, police or army
PEAS: “Plan Esencial de Aseguramiento Universal”
ENDES: “Encuesta Nacional Demográfica y de Salud Familiar”
INEI: “Instituto Nacional de Estadística e Informática”
ANC: Antenatal care
EPS: “Empresa Prestadora de Servicios”
PHI: Private health insurance
SD: Standard deviation
cOR: Crude odds-ratio
aOR: Adjusted odds-ratio
