# Supplementary material for: Comprehensive Health Insurance and access to maternal healthcare services among Peruvian women: a cross-sectional study using the 2021 national demographic survey
Source: BMC Pregnancy Childbirth. 2023 Nov 15;23:795. doi: 10.1186/s12884-023-06086-3 (PMC10647135; doi:10.1186/s12884-023-06086-3)
Supplement: Supplementary file 1 — Additional file 1. Appendix A. Variables’ categorization. Appendix B. Disaggregated bivariate analysis for “Effective ANC- prevention, -education and -screening”. Appendix C. Logistic regression for “Six ANC visits” and ANC 6 and “Skilled attendance at birth”. Appendix D. Logistic regression for “Effective ANC prevention” and “Effective ANC education”. Appendix E. Logistic regression for “Effective ANC screening”. [file 12884_2023_6086_MOESM1_ESM.docx]

| Appendix A: Variables’ categorization | | |
| --- | --- | --- |
| **Variable category** | **Variable** | **Categorization** |
| *Outcome variable* | **Six ANC visits** | 0 = Up to 5 ANC visits  1 = 6 or more ANC visits |
|  | **Skilled attendance at birth** | 0 = Unskilled attendance  1 = Skilled attendance |
|  | **Effective ANC prevention**  (Women attended at least six ANC + Received iron during pregnancy + Proper protection against Tetanus during pregnancy) | 0 = No  1 = Yes |
|  | **Effective ANC education**  (Women attended at least six ANC + received advice about pregnancy complications during any ANC) | 0 = No  1 = Yes |
|  | **Effective ANC screening**  (Women attended at least six ANC + Received all six antenatal care services during ANC visits) | 0 = No  1 = Yes |
| *Explanatory variable* | **Health Insurance Status** | 0 = No Insurance  1 = SIS  2 = Standard Insurance (EsSalud, EPS, FFAA/Police, PHI) |
| *Covariates* | **Age** | *Continuous* |
|  | **Highest educational level achieved** | 0 = Primary or no education  1 = Secondary  2 = Higher but no university  3 = Higher, university or PostGrade |
|  | **Ethnicity** | 0 = Other  1 = Spanish |
|  | **Marital status** | 0 = Not currently married  1 = Currently married |
|  | **Type of place of residence** | 0 = Rural  1 = Urban |
|  | **Ever had a terminated pregnancy** | 0 = No  1 = Yes |
|  | **Births in the last five years** | 0 = 1 birth  1 = 2 or more births |
|  | **Total children ever born** | 0 = 1 child  1 = 2 children  2 = 3 children  3 = 4 or more children |
|  | **Total living children** | 0 = 0-2 children  1 = 3 or more children |
|  | **Wealth index** | 0 = 1st quintile (poorest)  1 = 2nd quintile  2 = 3rd quintile  3 = 4th quintile  4 = 5th quintile (wealthiest) |
|  | **Household size** | 0 = 1-3 members  1 = 4-6 members  2 = 7 or more members |
|  | **Region** | (cluster variable) |

| Appendix B: Disaggregated bivariate analysis for “Effective ANC- prevention, -education and -screening” | | | | | | | | | |
| --- | --- | --- | --- | --- | --- | --- | --- | --- | --- |
| Variable | **Six ANC visits** | | | **Mother received advice about pregnancy complications** | | | **Mother received iron during pregnancy** | | |
| **Health Insurance Status** | **No**  **N (% row)** | **Yes**  **N (% row)** | **p** | **No**  **N (% row)** | **Yes**  **N (% row)** | **p** | **No**  **N (% row)** | **Yes**  **N (% row)** | **p** |
| *No insurance*  *SIS*  *Standard Insurance* | 436 (17.2)  1991 (14.9)  356 (10.7) | 2102 (82.8)  11327 (85.1)  2969 (89.3) | <0.001 | 490 (19.3)  1519 (11.4)  419 (12.6) | 2048 (80.7)  11799 (88.6)  2906 (87.4) | <0.001 | 190 (7.5)  674 (5.1)  141 (4.2) | 2348 (92.5)  12644 (94.9)  3184 (95.8) | <0.001 |
|  | **Mother was adequately vaccinated against Tetanus according to WHO** | | | **A minimum of six essential clinical measures were carried out at least once during the course of the ANC** | | |  | | |
|  | **No**  **N (% row)** | **Yes**  **N (% row)** | **p** | **No**  **N (% row)** | **Yes**  **N (% row)** | **p** |  |  |  |
| *No insurance*  *SIS*  *Standard Insurance* | 1350 (53.2)  6139 (46.1)  1639 (49.3) | 1188 (46.8)  7179 (53.9)  1686 (50.7) | <0.001 | 437 (17.2)  1639 (12.3)  375 (11.3) | 2101 (82.8)  11679 (87.7)  2950 (88.7) | <0.001 |  |  |  |

| Appendix C: Logistic regression for “Six ANC visits” and ANC 6 and “Skilled attendance at birth” | | | | |
| --- | --- | --- | --- | --- |
| **Variable** | **Six ANC visits** | | **Skilled attendance at birth** | |
|  | OR (95% CI) | p-value | OR (95% CI) | p-value |
| *Health Insurance Scheme* |  |  |  |  |
| No Insurance  SIS  Other insurance | Ref.  1.40 (1.14-1.73)  1.35 (1.13-1.63) | Ref.  <0.01  <0.01 | Ref.  2.12 (1.41-3.17)  2.17 (1.33-3.55) | Ref.  <0.01  <0.01 |
| *Highest educational level achieved* |  |  |  |  |
| Primary  Secondary  Higher, not university  Higher. University or postgrade | Ref  1.11 (0.94-1.31)  1.12 (0.90-1.40)  1.33 (1.02-1.74) | Ref.  0.24  0.30  0.04 | Ref.  2.16 (1.80-2.59)  4.07 (2.42-6.84)  3.38 (1.54-7.40) | Ref.  <0.01  <0.01  <0.01 |
| *Ethnicity* |  |  |  |  |
| Other  Spanish | Ref.  1.05 (0.88-1.25) | Ref.  0.50 | Ref.  1.00 (0.53-1.89) | Ref.  1.00 |
| *Marital status* |  |  |  | 0.000 |
| Not married  Married | Ref.  1.46 (1.30-1.63) | Ref.  <0.01 | Ref.  0.98 (0.81-1.20) | Ref.  0.87 |
| *Type of place of residence* |  |  |  |  |
| Rural  Urban | Ref.  0.70 (0.59-0.52) | Ref.  <0.01 | Ref.  2.53 (1.63-3.93) | Ref.  <0.01 |
| *Ever had a terminated pregnancy* |  |  |  |  |
| No  Yes | Ref.  0.92 (0.80-1.07) | Ref.  0.29 | Ref.  1.22(0.89-1.67) | Ref.  0.21 |
| *Births in last five years* |  |  |  |  |
| 1  2+ | Ref.  0.43 (0.39-0.47) | Ref.  <0.01 | Ref  0.65 (0.53-0.81) | Ref.  <0.01 |
| *Total children ever born* |  |  |  |  |
| 1 child  2 children  3 children  4 or more children | Ref  0.98 (0.77-1.23)  0.78 (0.50-1.20)  0.60 (0.37-0.96) | Ref.  0.82  0.25  0.04 | Ref.  0.57 (0.42-0.78)  0.37 (0.22-0.61)  0.19 (0.11-0.32) | Ref.  <0.01  <0.01  <0.01 |
| *Total living children* |  |  |  |  |
| 0-2  3+ | Ref.  0.96 (0.63-1.47) | Ref.  0.86 | Ref.  1.11 (0.67-1.81) | Ref.  0.69 |
| *Wealth index* |  |  |  |  |
| Poorest  Poor  Middle  Richer  Richest | Ref.  1.22 (0.95-1.56)  1.33 (1.08-1.63)  1.51 (1.08-2.12)  1.94 (1.41-2.66) | Ref.  0.12  0.01  0.02  <0.01 | Ref.  3.68 (2.58-5.27)  8.36 (4.49-15.56)  6.75 (3.90-11.67)  43.73 (4.52-423.46) | Ref.  <0.01  <0.01  <0.01  <0.01 |
| *Household size* |  |  |  |  |
| 1-3  4-6  7+ | Ref.  0.98 (0.87-1.11)  0.71 (0.59-0.84) | Ref.  0.74  <0.01 | Ref.  1.00 (0.83-1.22)  0.58 (0.45-0.73) | Ref.  0.96  <0.01 |
| *Age* | 1.04 (1.02-1.05) | <0.01 | 1.08 (1.06-1.09) | <0.01 |
| *Constant* | 1.54 (0.89-2.66) | 0.12 | 0.64 (0.21-1.94) | 0.42 |

| Appendix D: Logistic regression for “Effective ANC prevention” and “Effective ANC education” | | | | |
| --- | --- | --- | --- | --- |
| Variable | **Effective ANC prevention** | | **Effective ANC education** | |
|  | OR (95% CI) | p-value | OR (95% CI) | p-value |
| *Health Insurance Scheme* |  |  |  |  |
| No Insurance  SIS  Other insurance | Ref.  1.32 (1.16-1.50)  1.22 (1.04-1.42) | Ref.  <0.01  0.01 | Ref.  1.59 (1.41-1.80)  1.34 (1.18-1.51) | Ref.  <0.01  <0.01 |
| *Highest educational level achieved* |  |  |  |  |
| Primary  Secondary  Higher, not university  Higher. University or postgrade | Ref  0.96 (0.84-1.10)  0.93 (0.78-1.12)  0.84 (0.67-1.07) | Ref.  0.57  0.47  0.15 | Ref.  1.03 (0.88-1.20)  1.08 (0.89-1.30)  1.19 (0.96-1.47) | Ref.  0.71  0.44  0.11 |
| *Ethnicity* |  |  |  |  |
| Other  Spanish | Ref.  3.76 (2.50-5.64) | Ref.  <0.01 | Ref.  8.12 (5.01-13.17) | Ref.  <0.01 |
| *Marital status* |  |  |  |  |
| Not married  Married | Ref.  1.22 (1.13-1.32) | Ref.  <0.01 | Ref.  0.57 (0.47-0.68) | Ref.  <0.01 |
| *Type of place of residence* |  |  |  |  |
| Rural  Urban | Ref.  0.84 (0.72-0.99) | Ref.  0.04 | Ref.  2.53 (1.63-3.93) | Ref.  <0.01 |
| *Ever had a terminated pregnancy* |  |  |  |  |
| No  Yes | Ref.  0.93 (0.82-1.04) | Ref.  0.20 | Ref.  0.96 (0.86-1.07) | Ref.  0.49 |
| *Births in last five years* |  |  |  |  |
| 1  2*+* | Ref.  0.56 (0.50-0.63) | Ref.  <0.01 | Ref  0.54 (0.50-0.59) | Ref.  <0.01 |
| *Total children ever born* |  |  |  |  |
| 1 child  2 children  3 children  4 or more children | Ref  0.96 (0.87-1.06)  0.91 (0.58-1.43)  0.88 (0.53-1.47) | Ref.  0.44  0.69  0.63 | Ref.  0.98 (0.86-1.12)  0.96 (0.69-1.33)  0.83 (0.55-1.25) | Ref.  0.75  0.81  0.37 |
| *Total living children* |  |  |  |  |
| 0-2  3+ | Ref.  1.03 (0.69-1.53) | Ref.  0.90 | Ref.  0.88 (0.61-1.29) | Ref.  0.52 |
| *Wealth index* |  |  |  |  |
| Poorest  Poor  Middle  Richer  Richest | Ref.  0.99 (0.84-1.15)  0.99 (0.80-1.22)  1.02 (0.77-1.35)  0.86 (0.62-1.19) | Ref.  0.85  0.92  0.89  0.37 | Ref.  0.88 (0.70-1.11)  0.86 (0.70-1.08)  0.95 (0.71-1.28)  1.09 (0.85-1.41) | Ref.  0.29  0.19  0.75  0.49 |
| *Household size* |  |  |  |  |
| 1-3  4-6  7+ | Ref.  1.11 (1.00-1.23)  1.19 (1.00-1.42) | Ref.  0.04  0.05 | Ref.  1.03 (0.91-1.16)  0.80 (0.69-0.97) | Ref.  0.68  0.02 |
| *Age* | 1.01 (1.00-1.02) | 0.03 | 1.02 (1.02-1.03) | <0.01 |
| *Constant* | 0.18 (0.11-0.29) | <0.01 | 0.31 (0.19-0.50) | <0.01 |

| Appendix E: Logistic regression for “Effective ANC screening” | | |
| --- | --- | --- |
| Variable | **Effective ANC screening** | |
|  | OR (95% CI) | p-value |
| *Health Insurance Scheme* |  |  |
| No Insurance  SIS  Other insurance | Ref.  1.47 (1.27-1.69)  1.31 (1.15-1.49) | Ref.  <0.01  <0.01 |
| *Highest educational level achieved* |  |  |
| Primary  Secondary  Higher, not university  Higher. University or postgrade | Ref  1.03 (0.87-1.22)  1.08 (0.88-1.33)  1.23 (0.99-1.54) | Ref.  0.75  0.47  0.06 |
| *Ethnicity* |  |  |
| Other  Spanish | Ref.  8.25 (4.92-13.84) | Ref.  <0.01 |
| *Marital status* |  |  |
| Not married  Married | Ref.  1.38 (1.28-1.49) | Ref.  <0.01 |
| *Type of place of residence* |  |  |
| Rural  Urban | Ref.  0.64 (0.52-0.78) | Ref.  <0.01 |
|  |  |  |
| *Ever had a terminated pregnancy* |  |  |
| No  Yes | Ref.  0.99 (0.89-1.10) | Ref.  0.86 |
| *Births in last five years* |  |  |
| 1  2+ | Ref.  0.51 (0.46-0.56) | Ref.  <0.01 |
| *Total children ever born* |  |  |
| 1 child  2 children  3 children  4 or more children | Ref  1.01 (0.89-1.15)  1.04 (0.72-1.52)  0.88 (0.59-1.32) | Ref.  0.85  0.83  0.54 |
| *Total living children* |  |  |
| 0-2  3+ | Ref.  0.87 (0.59-1.28) | Ref.  0.48 |
| *Wealth index* |  |  |
| Poorest  Poor  Middle  Richer  Richest | Ref.  0.94 (0.73-1.20)  0.93 (0.77-1.14)  1.07 (0.76-1.49)  1.11 (0.82-1.51) | Ref.  0.59  0.51  0.71  0.48 |
| *Household size* |  |  |
| 1-3  4-6  7+ | Ref.  1.03 (0.91-1.17)  0.81 (0.68-0.96) | Ref.  0.67  0.01 |
| *Age* | 1.02 (1.02-1.03) | <0.01 |
| *Constant* | 0.26 (0.15-0.44) | <0.01 |
